# Formulation of perfect-crystal diffraction from Takagi–Taupin equations: numerical implementation in the *crystalpy* library

**DOI:** 10.1107/S160057752400924X

**Published:** 2024-10-29

**Authors:** Jean-Pierre Guigay, Manuel Sanchez del Rio

**Affiliations:** a European Synchrotron Radiation Facility 71 Avenue des Martyrs F-38000Grenoble France; Brazilian Synchrotron Light Laboratory, Brazil

**Keywords:** X-ray diffraction, perfect crystal, dynamical theory of diffraction, *crystalpy* software package, crystal optics

## Abstract

The Takagi–Taupin equations are solved in their simplest form (zero deformation) to obtain the Bragg-diffracted and transmitted complex amplitudes. The case of plane-parallel crystal plates is discussed using a matrix model. The equations are implemented in an open-source Python library *crystalpy* adapted for numerical applications such as crystal reflectivity calculations and ray tracing.

## Abstract 2

The Takagi–Taupin equations are solved in their simplest form (zero deformation) and equations of the diffracted and transmitted amplitudes are obtained using a matrix model. The theory presented is coded in the open-source software package *crystalpy*.

## 1. Introduction

Almost every synchrotron radiation beamline operating with hard X-rays makes use of perfect crystals. Most beamlines use a double-crystal monochromator with flat crystals. Multiple reflections are used for high resolution (Ishikawa *et al.*, 2005 ▸; Shvyd’ko, 2004 ▸). Curved crystals are used in reflection [polychromators for dispersive X-ray absorption spectroscopy (Tolentino *et al.*, 1988 ▸)] or in transmission [single- (Suortti *et al.*, 1993 ▸) or double-crystal Laue monochromators (Ren *et al.*, 1999 ▸)]. Plane crystals plates are used to change the polarization state of X-rays (Bouchenoire *et al.*, 2003 ▸; Detlefs *et al.*, 2012 ▸). In addition, crystal analyzers are used in most spectroscopy beamlines [see, for example, Rovezzi *et al.* (2017 ▸)].

Beamline simulation tools used for the design, optimization and commissioning of synchrotron instrumentation implement in software the equations to calculate the reflectivity of perfect crystals. The theory of diffraction [see Authier (2003 ▸) for a complete reference] is the basis of all numeric implementations.

There are many simulation tools implementing the equations of the dynamical theory in different forms. This variate scenario is even more complex if we consider that the calculation of the crystal structure factor, which is an essential ingredient for calculating diffracted amplitudes and intensities, is obtained from tabulated scattering functions of multiple origins. A wide collection of software methods and tools can be found even in a single application, such as the *OASYS* suite (Rebuffi & Sanchez del Rio, 2017 ▸), which provides multiple solutions for calculating diffraction profiles of crystals [*e.g.INPRO* (https://github.com/oasys-kit/xoppy_external_codes/tree/master/src/INPRO), *CRYSTAL* (Sanchez del Rio *et al.*, 2015 ▸), *X-RAY Server* (Stepanov, 2004 ▸)], as well as beamline simulation tools [based on the ray-tracing code *SHADOW* (Sanchez del Rio *et al.*, 2011 ▸)] and physical wave-optics simulations with *SRW* (Chubar & Elleaume, 1998 ▸; Sutter *et al.*, 2014 ▸). Most ray-tracing codes used for synchrotron applications incorporate models for crystal diffraction, like *SHADOW* (Sanchez del Rio *et al.*, 2011 ▸), *RAY* (Schäfers, 2008 ▸; Baumgärtel *et al.*, 2019 ▸), *XRT* (Chernikov & Klementiev, 2017 ▸; Klementiev & Chernikov, 2023 ▸). This scenario has inherited decades of advancements and has witnessed the evolution of several generations of synchrotron radiation sources. Our research aims to tackle this challenge by consolidating the resources for crystal diffraction calculations. We have two primary objectives: deducing the equations governing crystal reflectivity from first principles and integrating them into a thoroughly documented open-source software library.

The Takagi–Taupin equations are a powerful tool in Bragg diffraction by deformed crystals for diverse forms of the incident monochromatic wave. They are applied here to the simple particular case of plane parallel crystals and plane incident waves. We derive results found in the conventional dynamical theory described in textbooks (Zachariasen, 1994 ▸; Pinsker, 1978 ▸; Authier, 2003 ▸). Therefore, there are no new physical results in the present paper. However, the method presented here is mathematically well defined and simple. It is general in the sense that it deals directly with absorbing crystals. We believe that it represents a valuable and useful shortcut to the conventional method.

In Section 2 we derive the Takagi–Taupin (TT) equations (Takagi, 1962 ▸; Taupin, 1964 ▸; Taupin, 1967 ▸). In Section 3 we solve the TT equations for a plane undeformed-crystal. Given known complex amplitudes at the entrance surface, the complex amplitudes along the incident and diffracted directions at the back surface are calculated via a transfer matrix (Section 3.2). For the Laue case, the transfer matrix is directly used to compute the diffracted and forward-diffracted (or transmitted) complex amplitudes (Section 3.4). For the Bragg case (Section 3.5) the transfer matrix is used to obtain the scattering matrix, which gives the diffracted and transmitted complex amplitudes. Section 4 is dedicated to the software implementation of the library *crystalpy*. A final summary and conclusions are in Section 5.

## 2. Takagi–Taupin equations

The scalar time-independent X-ray wave equation in a perfect crystal is the Helmholtz equation,
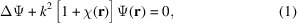
where 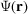 is the wavefunction, *k* = 2π/λ, with λ the wavelength, 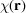 is the electric susceptibility [refractive index *n* = (1 + χ)^1/2^] that can be expanded in a Fourier series, 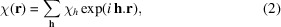
where the sum goes over all reciprocal lattice vectors **h** with (*hkl*) Miller indices. The spacing of the (*hkl*) reflection is *d*_*hkl*_ = 2π/*h*, where *h* = 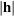.

Let us consider an incident plane wave 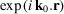 in vacuum. Its wavevector 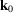, with modulus *k* = 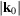, is close to the Bragg condition for the diffraction vector **h**. In the ‘two-beams case’ of Bragg diffraction, considered in this paper, we define 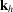

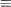

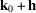
, of modulus 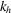 = 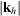. In general, the direction of 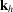 does not correspond to the Bragg-diffracted wavevector in vacuum, and *k*_*h*_ is slightly different from *k*. The deviation from the exact Bragg position is expressed by the parameter α (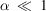), defined as 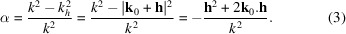
The wavefield 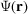 in the crystal is set empirically as the sum of ‘two modulated plane waves’,
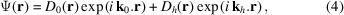
in which the amplitudes 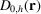 are considered as ‘slowly varying functions’, thus neglecting their second-order derivatives in 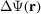, 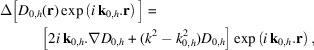
thus giving 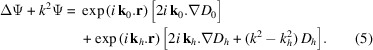
In the product 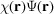, using the equations (2) and (4), we write separately the terms containing either 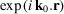 or 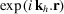, and do not consider the other terms, 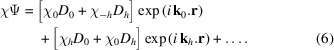
Inserting equations (5) and (6) into (1), we obtain the TT equations, 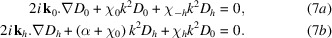
We can use the oblique coordinates (*s*_0_, *s*_*h*_) in the diffraction plane (the plane containing 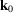 and **h**, as well as 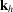), with origin *O* on the crystal surface and unit vectors 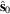 and 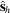 along 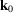 and 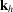, respectively. A generic spatial position 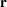 = 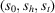 should include a third coordinate *s*_*t*_ along an axis 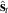 non-coplanar with 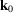 and 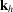. We can choose 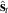 to lie on the crystal entrance surface and be perpendicular to the intersection line of the diffraction plane and the crystal surface. The chosen direction of 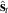 implies 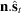 = 0. The equation of the crystal surface is γ_0_*s*_0_ + γ_*h*_*s*_*h*_ = 0, with 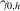 = 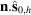

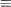

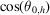
 the director cosines with respect to **n**, the unit inward normal vector to the entrance plane surface.

The relation 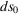 = 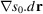 = 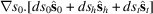 implies 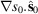 = 1 and 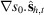 = 0. Similarly, 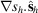 = 1 and 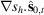 = 0. Therefore, 
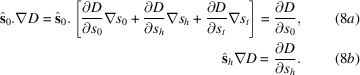
Using the approximation^1^
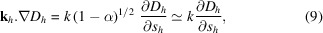
we obtain from equation (7),
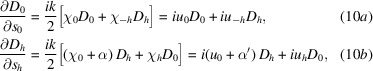
where we used the notation 
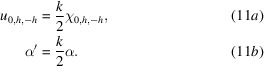
Our formulation is written for the case of σ-polarization. For the case of π-polarization, it is sufficient to replace χ_*h*_ and χ_−*h*_ with *C*χ_*h*_ and *C*χ_−*h*_ with *C* = 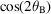. An equivalent form of the TT equations (10) is obtained in Appendix *A*[App appa], using oblique axes along the directions of the geometrical Bragg law, and applying a crystal rotation.

## 3. Solutions of TT equations for a plane wave incident on a crystal with plane entrance surface

It is interesting to consider first the effects of refraction and absorption without Bragg diffraction. Setting *u*_−*h*_ = 0 in equations (10*a*), we obtain the following equation for the refracted amplitude,
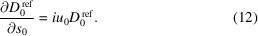
Its solution satisfying the boundary conditions 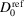 = 1 for γ_0_*s*_0_ + γ_*h*_*s*_*h*_ = 0 (equation of the crystal surface) is 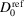 = 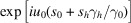 = 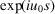 where 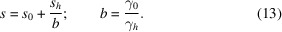
We now consider the solutions of the equations (10) depending on the single variable^2^*s*, which means ∂*D*_0_/∂*s*_0_ = 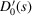 and ∂*D*_*h*_/∂*s*_*h*_ = 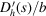. The equations (10) become 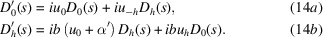
It is convenient to use the functions *B*_0,*h*_(*s*) by setting 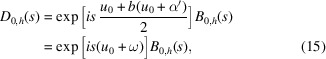
with 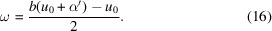
Equations (14) become 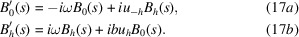
They have special solutions of the form^3^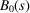 = 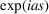 and 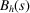 = 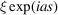, which, introduced in equation (17), give ξ = *bu*_*h*_/(*a* − ω) = (*a* + ω)/*u*_−*h*_ and 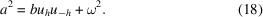
The general solution of equation (17) is
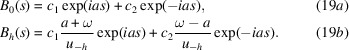
From the case *s* = 0, we obtain 
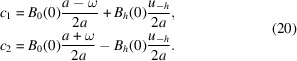
Consequently 
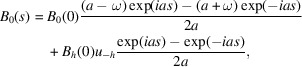

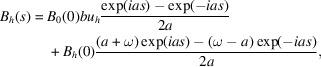
or, in terms of *D*_0,*h*_(*s*) [equation (15)] in a more compact form, 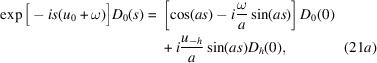

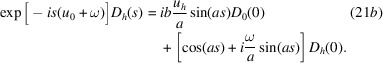


### Expressions of α, ω and 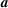 as functions of the angles

3.1.

Note that *h* = 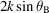, θ_B_ being the geometrical Bragg angle, and 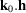 = 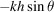, with θ the glancing angle of 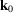 on the reflecting planes. From equation (3) we obtain 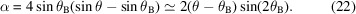
Our definition of α [equation (3)] was made in such a way that α increases when θ increases.^4^ The approximated value of α is not valid far from the Bragg position or when θ_B_ approaches π/2 (normal incidence); therefore equation (3) is used in the *crystalpy* software.

α′ [equation (11)] and ω [equation (16)] are 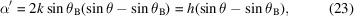

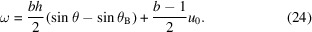
The ‘corrected Bragg angle’ θ_c_, that differs from θ_B_ because of the effect of refraction, is obtained as the θ value such that 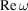 = 0, or 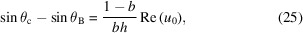
which, under the usual conditions [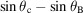 ≃ 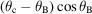], reduces to 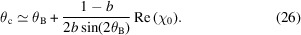
From equations (24) and (25), the value of ω has a simple expression as a function of θ_c_ and θ, 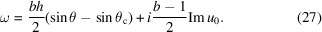
Note that, in our representation [using waves of the form 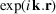], we have 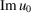 ≥ 0. Equations (21) are expressed in terms of *a*, but they depend only on *a*^2^. Using equation (27) in equation (18) we obtain 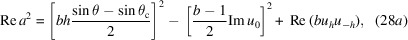

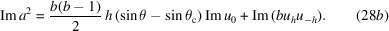
Note that *a* can be expressed in terms of *a*^2^ as 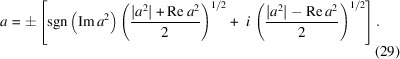


### 3.2. The transfer matrix of a parallel crystal slab

The front and back surfaces of a crystal parallel slab correspond to *s* = 0 and *s* = *t*_c_/γ_0_ = *T*, respectively, with *t*_c_ the ‘usual’ thickness of the crystal. We can express the fields at the back surface (*D*_0_(*T*), *D*_*h*_(*T*)) in terms of those at the front surface (*D*_0_(0), *D*_*h*_(0)) in matrix form, 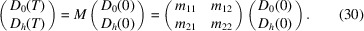
According to equation (21), the elements of the ‘transfer matrix’ *M* are 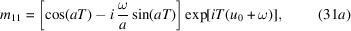

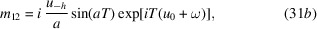

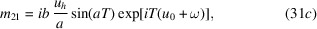

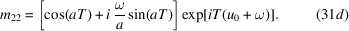
The determinant of the matrix *M* is 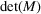 = 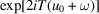. Its modulus |det(*M*)| ≤ 1. It is 1 for a non-absorbing crystal (*u*_0_ and ω are real). This is in agreement with the expected energy conservation. It can be verified that *M*(*T*_1_ + *T*_2_) = *M*(*T*_2_)*M*(*T*_1_) and *M*(−*T*) = [*M*(*T*)]^−1^. Last, but not least, equation (30) is valid for both Bragg and Laue cases (with adequate values of *b*, *a* and ω).

### 3.3. The transfer matrix for the case of a ‘thick crystal’

Equations (21) and (31) are expressed in terms of *a*, but they depend only on *a*^2^. It is possible to write them as 
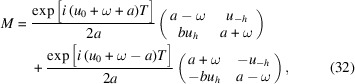
where the two terms are interchanged when *a* is changed in −*a*. They correspond to the two roots of *a*^2^. They also correspond to the two branches of the dispersion surface [see, for example, Authier (2003 ▸)]. The real part of the argument of the exponential factors, 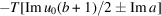, is related to the absorption. When 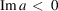, the absorption is less than 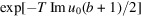 for the first term, and more than that for the second one. Similarly, for 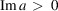 the two matrices present opposite behaviour. If 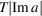 is large (for example 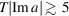), we can keep only the largest term in equation (32),
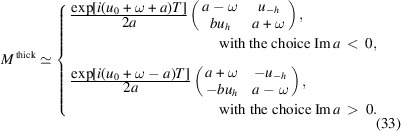
A beam, that would be absorbed without Bragg diffraction if 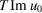

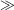
 1 may partially go through the ‘thick crystal’ in the condition of Bragg diffraction. Equation (33) is a clear expression of the Borrmann effect.

### 3.4. Reflection and transmission amplitudes in the Laue case

**Figure 1:**
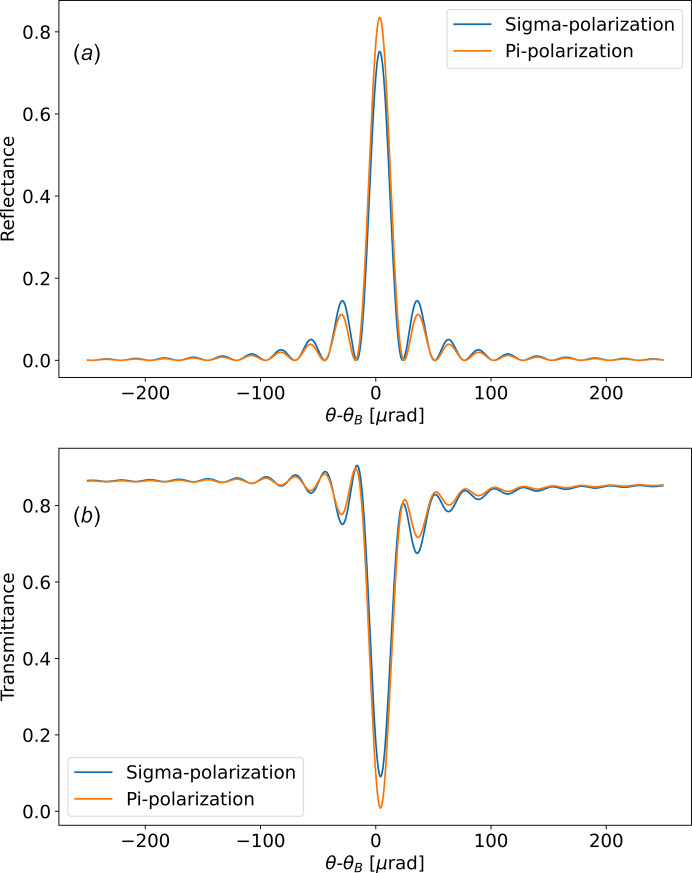
Calculated reflectance (*a*) and transmittance (*b*) for a 10 µm-thick Laue Si 111 crystal at 8 keV, with 65° of asymmetric angle. The Bragg angle is θ_B_ = 14.31°.

In this case, *b* > 0. The boundary conditions are (*D*_0_(0), *D*_*h*_(0)) = (1, 0). The reflection and transmission amplitudes, *r*_L_ = *D*_*h*_(*T*) and *t*_L_ = *D*_0_(*T*), respectively, are directly written from the matrix equation (30),
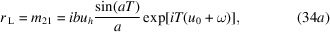

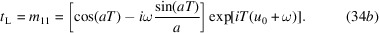
The reflecting power is 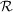 = 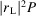, where *P* = |*b*|^−1^ is the ratio of the cross sections of the reflected and incident beams (Zachariasen, 1994 ▸). Its plot 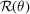 as a function of the angle of incidence is the diffraction profile, often referred to as the rocking curve. 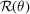 and 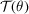 = 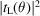 are hereafter called reflectance and transmittance, respectively. An example is shown in Fig. 1 ▸.

It is also interesting to consider the case of incidence along the direction of 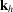 (diffraction vector 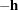), for which (*D*_0_(0), *D*_*h*_(0)) = (0, 1). It is directly seen from equation (30) that the transmission and reflection amplitudes are 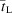 = 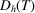 = 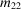 and 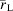 = 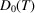 = 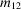 (note that the reflection power factor is *P* = |*b*| in this case). These results can be written as 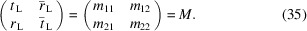
This means that, for the Laue case, the matrix *M* can be considered not only as the ‘transfer-matrix’ of the crystal slab but also as the ‘scattering-matrix’ (*S*-matrix) which relates the vacuum waves leaving the crystal to the vacuum waves entering it, in analogy with the *S*-matrix used in general scattering theory.

The exponential factor in equation (34) gives a damping factor, which is {using *u*_0_ + ω = [(*b* + 1)*u*_0_ + *b*α′]/2 from equation (16), and noting that α′ is real} 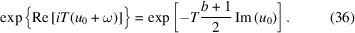
The Pendellösung effect is due to the oscillations of 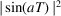 = 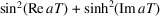. The Pendellösung distance (depending on θ) along *s*_0_ is thus equal to 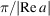 = 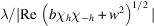, where *w* = (λ/π)ω. At θ = θ_c_, 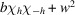 = 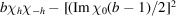. In the symmetric Laue case (*b* = 1) we obtain the well known formula of the Pendellösung distance along the direction normal to the crystal surface [see, for example, equation (3.48) of Pinsker (1978 ▸)], 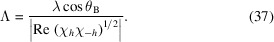


### 3.5. Reflection and transmission amplitudes in the Bragg case – the S-matrix

**Figure 2:**
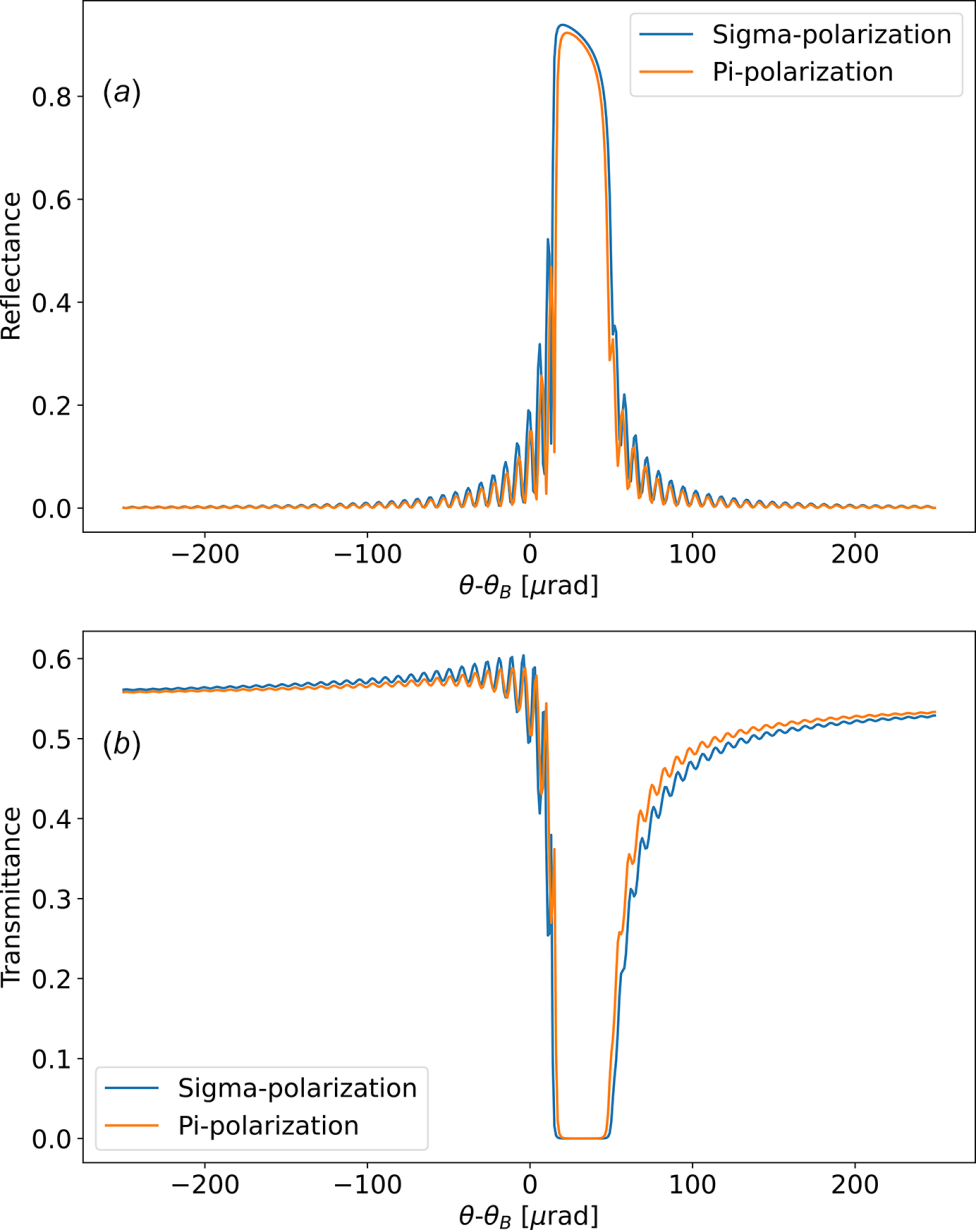
Calculated reflectance (*a*) and transmittance (*b*) of a symmetrical Bragg Si 111 crystal with 10 µm thickness at 8 keV.

In this case, *b* < 0. We set *D*_0_(0) = 1 and *D*_*h*_(*T*) = 0 (which means no beam entering the crystal slab on the back surface). The reflection and transmission amplitudes are *r*_B_ = *D*_*h*_(0) and *t*_B_ = *D*_0_(*T*), respectively. Equation (30) is 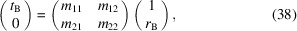
from which we obtain 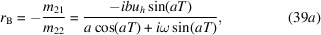

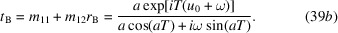
These solutions, as well as those for Laue in equations (34), can also be obtained by direct integration of the TT equations (17) using Laplace transforms (see Appendix *B*[App appb]). Similarly, in the case of incidence on the crystal back side along the direction 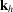 (diffraction vector 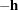), we set *D*_*h*_(*T*) = 1, 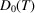 = 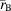, *D*_0_(0) = 0 and 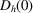 = 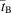. Therefore, equation (30) gives 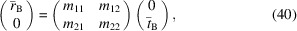
from which we obtain 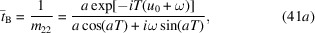

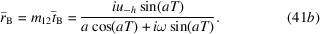
Consequently, the *S*-matrix for the Bragg case, defined as 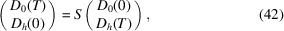
is 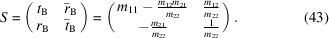
The diffraction profile (reflectance) is 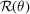 = 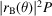, with *P* = 1/|*b*|, and the transmittance is 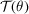 = 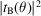. An example is shown in Fig. 2 ▸.

**Figure 3:**
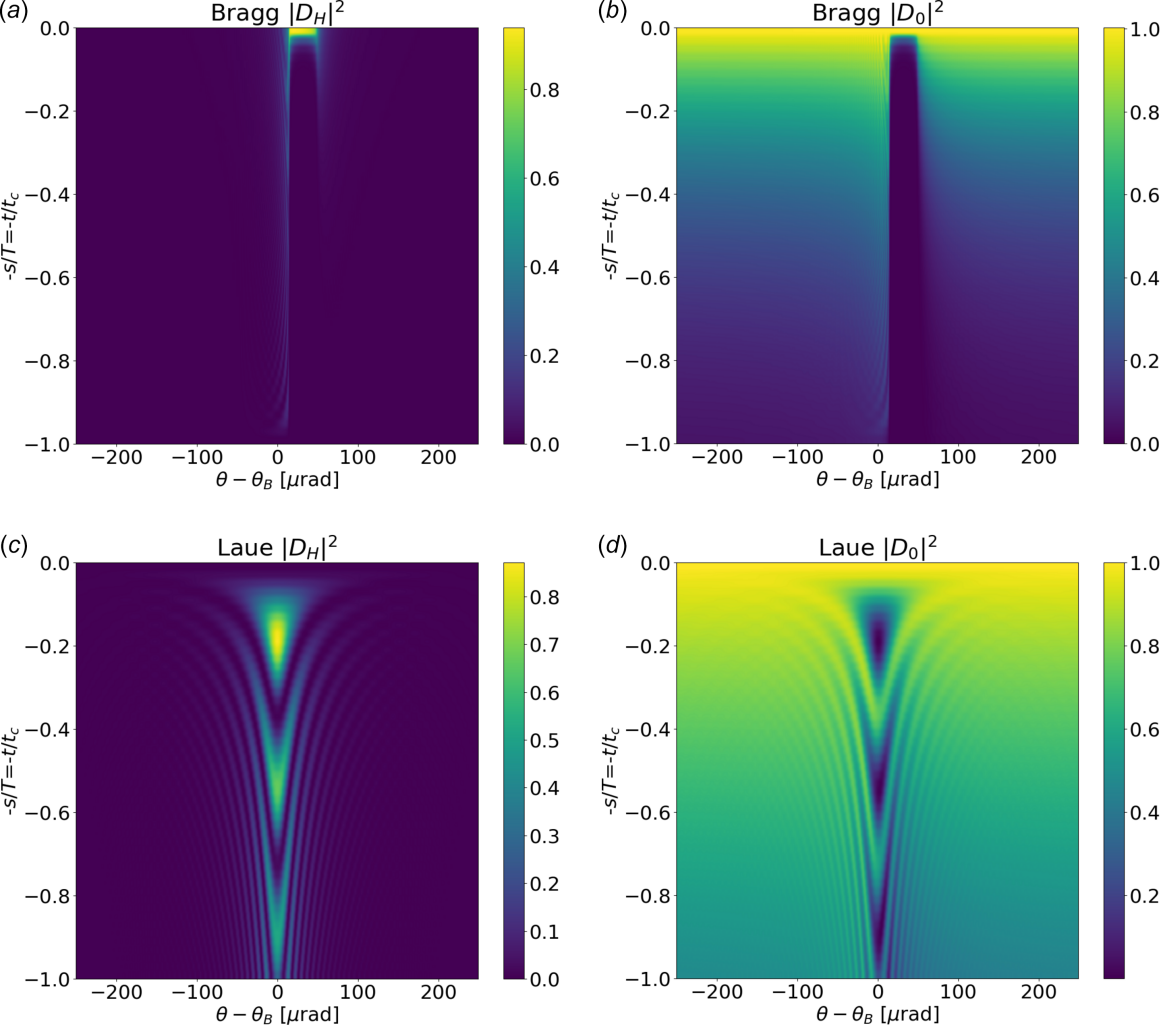
Calculations for a symmetric Si 111 at 8 keV with thickness *t*_c_ = 50 µm. The graphs show the electric field intensity inside the crystal as a function of the deviation angle θ − θ_B_ and penetration ratio −*s*/*T* (equivalent to a depth ratio − *t*/*t*_c_), for (*a*) Bragg |*D*_*h*_|^2^, (*b*) Bragg |*D*_0_|^2^, (*c*) Laue |*D*_*h*_|^2^, (*d*) Laue |*D*_0_|^2^.

The field inside the crystal, *i.e.D*_0_(*s*) and *D*_*h*_(*s*), can be calculated using equation (19), with *D*_0_(0) = 1 and *D*_*h*_(0) = *r*_B_ from equation (39*a*) we obtain 
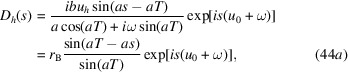

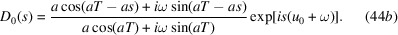
An example of simulation of the field inside the crystal using equation (44) is shown in Fig. 3 ▸. For the Laue case, also shown in this figure, we observe that the field at coordinate *s* is simply calculated by the equations (34) replacing *T* by *s*.

**Figure 4:**
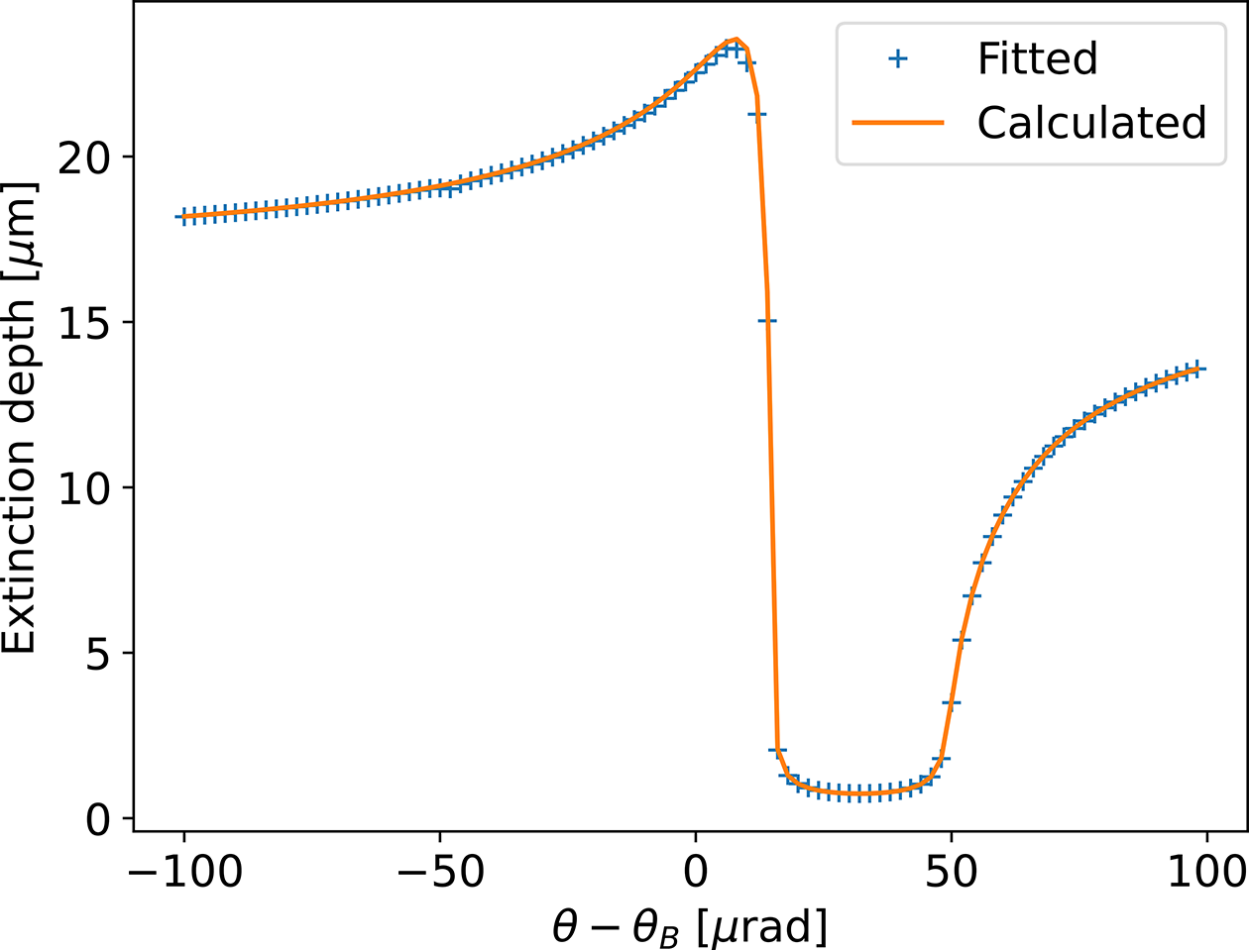
Fit of |*D*_0_|^2^ versus *t* of Fig. 3 ▸(*b*) with a function 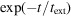 to obtain the extinction depth *t*_ext_. This result is compared with the calculated value from equation (45) that gives 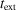 = 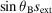 = 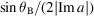.

Fig. 3 ▸(*b*) shows that the penetration of the incident wave inside the crystal is small in a limited interval around θ_c_. In Fig. 4 ▸ we fitted the intensity profile of |*D*_0_(*s*)|^2^ versus depth for each value of θ − θ_B_. The fact that for a thick crystal in Bragg geometry |*D*_0_(*s*)|^2^ has significant values only in the vicinity of the crystal surface, in the central region, can be explained from equation (44). The moduli of the functions 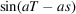 and 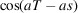 are approximately proportional to 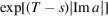 if the argument of this exponential function is sufficiently large. Consequently, |*D*_0_(*s*)|^2^ is nearly proportional to 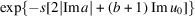. Writing 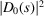 = 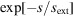, with 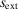 = 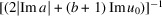 the extinction length (measured along the *s*_0_ axis), we obtain for the Bragg symmetric (*b* = −1) case, 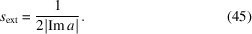


#### 3.5.1. Reflection amplitude for a thick absorbing crystal in the Bragg case

In the case of thick (or semi-infinite) Bragg crystal, the reflection amplitude, given by equation (39), takes the form [using *M* from equation (33)] 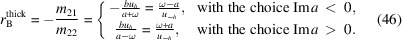
Both equations are equivalent assuming that the sign in *a* = ±(*a*^2^)^1/2^ is correctly selected. The condition on 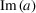 in equation (46) is in accordance with the physical condition that |*r*_B_| goes to zero when 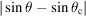 is large. The condition 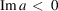 [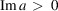] is equivalent^5^ to 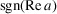 = 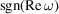 [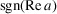 = 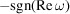] for large values of 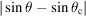.

Equation (46) is a useful result, as most crystal monochromators used in synchrotron radiation are thick crystals in Bragg (reflection) mode.

#### 3.5.2. Reflection amplitude for non-absorbing crystals in the Bragg case

In this case 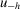 = 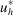; ω [see equation (16)] and 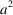 = 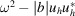 are real. We can distinguish two cases.

If *a*^2^ ≤ 0, or 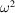

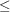

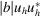
, then *a* = 
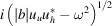
; therefore, according to equation (46), 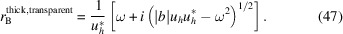
If *a*^2^ > 0, or 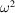 > 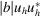, 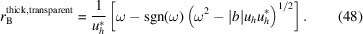
Equation (48) represents the tails of the reflection profile. As discussed previously, the sign selection is such that |*r*_B_| tends to zero for large values of |ω|. Equation (47) corresponds to the zone of total reflection. The reflectance is 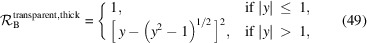
with *y* = 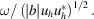


### 3.6. Calculation of reflection and transmission amplitudes using the transfer matrix

The matrix method permits the complex reflection and transmission amplitudes of a crystal made by layers of different crystals (or the same crystal with different orientations) to be obtained. For that, (i) construct the transfer matrix of the total crystal by multiplication^6^ of the transfer matrices of the different layers [each one calculated using equation (31)]; (ii) if the geometry is Laue, obtain reflection and transmission amplitudes using the coefficients *m*_21_ and *m*_11_, respectively, of this matrix [equation (34)]; otherwise (Bragg geometry), compute the scattering matrix using equation (43) and the reflection and transmission amplitudes are given in the matrix terms *s*_21_ and *s*_11_ [equation (39)], respectively.

**Figure 5:**
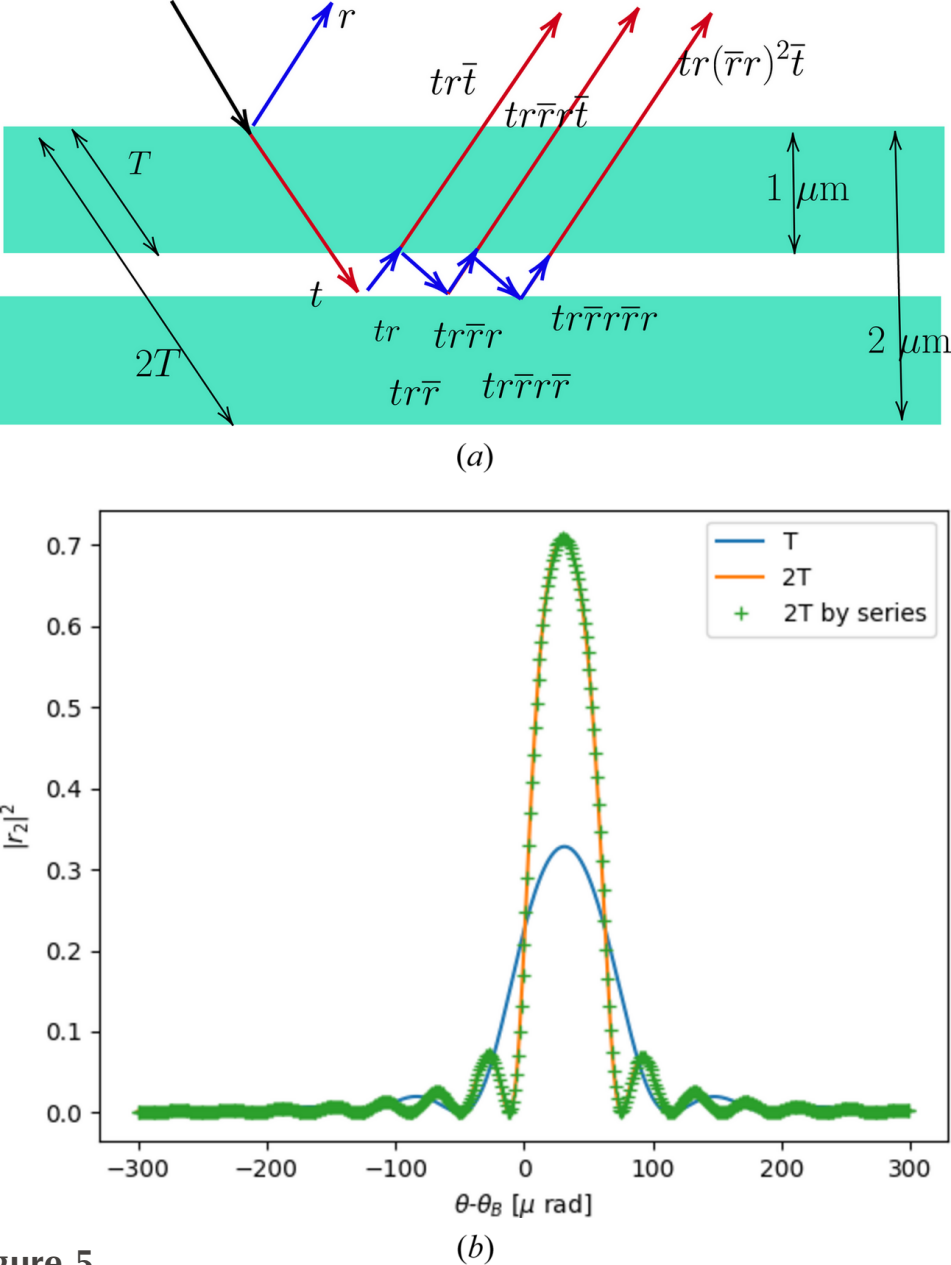
Example of calculation of the reflection amplitude *r*_2_ of a Si 111 crystal of 2 µm thickness from the amplitudes of the half-layer (1 µm). The reflectivity of the bilayer *r*_2_ can be obtained as an infinite sum 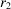 = 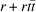 + 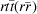 + 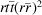 + … = 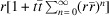 = 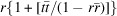. Calculations done with *crystalpy* for a photon energy of 8 keV.

A first example shows how simple is the application of this recipe of multiplication of transfer matrices to obtain the reflectance of a simple two-layer crystal. Consider a bilayer of two identical crystal layers of thickness *T* and transfer matrix *M* for each one. Using matrix analysis, the transfer matrix of the bilayer is [*M*(*T*)]^2^ = *M*(2*T*) from which it is easy to compute the reflectivity in Bragg geometry [equation (39)]. Otherwise, if this result would be obtained via the reflectivities (*r* and 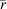) and transmittivities (*t* and 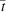) of the single layer (*S*-matrix), the reflectivity of the bilayer results from an infinite series as shown in Fig. 5 ▸.

A second example is the Bragg reflection of a crystal layer on a thick substrate. The transfer matrix is calculated as 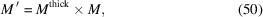
with *M*^thick^ the transfer matrix of the substrate and *M* the transfer matrix of the thin layer. We are interested in the Bragg reflectivity or 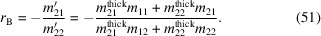
This can be expressed as a function of the substrate reflectivity 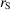 = 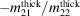 giving
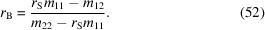
The method of transfer matrix multiplication can also be used for analysing distorted and bent crystals and will be explored in a future work.

### 3.7. The direction of the diffracted wave in vacuum

In some applications, as in ray tracing, it is essential to know the diffracted wavevector 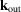 that exits from the crystal. As mentioned before, the choice of 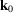 in equation (4) is somewhat arbitrary. In our choice, 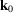 corresponds exactly to the wavevector of the incident plane wave. The vector 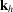 (see Section 2), defined as 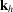 = 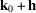, does not correspond in general to the wavevector of the outgoing ray or wave outside the crystal. 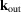 has the form 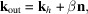
with **n** the unit vector along the inward normal to the crystal exit surface. The (real) coefficient β is obtained by writing that the modulus of 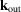 is equal to *k*,
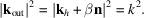
Note that 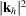 = 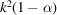 and 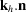 = 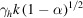, from which we obtain the equation 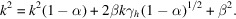
Its solutions are 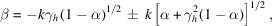
where the ± sign is chosen in such a way that β = 0 when α = 0 (*i.e.* positive for γ_*h*_ > 0 and negative for γ_*h*_ < 0).

## 4. The *crystalpy* library

*Crystalpy* is a Python library that performs calculations on diffraction from perfect crystals using the formalism introduced in the previous chapters.

The motivation of *crystalpy* was to create a modern, extensible, well documented and friendly library to overcome the difficulties of integrating ancient software tools based on the dynamical diffraction theory. It is specifically designed for two objectives: support for new versions of the crystal diffraction codes delivered in platforms like *OASYS* (Rebuffi & Sanchez del Rio, 2017 ▸), and to provide a core for ray-tracing simulations with crystals. The *crystalpy* library is written in the Python language and uses standard libraries (*NumPy* and *SciPy*). It makes use of vector calculus and stack operations to accelerate the calculations. Therefore, it is adapted for being used in ray-tracing tools, such as the future *SHADOW* (Sanchez del Rio *et al.*, 2011 ▸) versions.

To simulate a diffraction experiment using a perfect crystal, *crystalpy* offers functions that implement the theory described previously. Two input objects must be prepared: (i) the incident wave(s) or photon ray(s), and (ii) the information on the crystal (diffraction setup). The objects representing these two entities are described here.

The 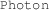 class is a minimum class for a photon, containing the energy (in eV) and a unit direction vector, implemented in the 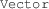 class. It deals with the storage and operations (addition, scalar product, cross product, normalization, rotation around an axis, *etc*.) of a 3D vector. A superclass of 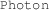 is 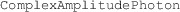, that contains the scalar complex amplitude for σ and π polarizations). These classes (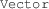, 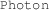 and 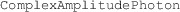) can hold stacks (the internal storage is done with arrays to speed-up vector operations). The 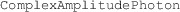 class has a corresponding 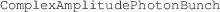 superclass, decorated with methods to deal with multiple waves or beams (bunches or sets of photons).

The information on the crystal itself (*e.g.* particular crystal material and crystal structure), its preparation (crystal cut) and related physical parameters (like the structure factor) are managed by the 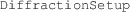 classes. *crystalpy* allows multiple options to retrieve the crystal structure and the scattering functions needed to calculate the structure factors. The 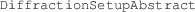 class defines the methods to access the basic information of the crystal (defined as a string, *e.g.* ‘Si’) such as 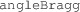, 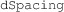 and 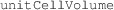, and to compute the structure factors: 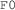, 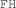, 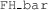. These parameters can be obtained from several libraries external to *crystalpy*. We implemented three options: (i) 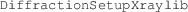 using the 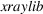 library (Schoonjans *et al.*, 2011 ▸), (ii) 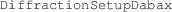 that uses the *dabax* library (Sanchez del Rio, 2021 ▸), and (iii) using an *ad hoc* generated data file. This modular structure permits disconnecting the calculation part from the access to optical and physical constants. Indeed, when using *ad hoc* data files we do not have to import 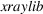 or *dabax* packages. We implemented this for the crystal material files of the *SHADOW* (Sanchez del Rio*et al.*, 2011 ▸) code in the traditional version (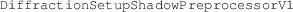), and in a version supporting *d*-spacing crystals (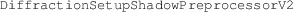). The 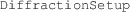 classes handle the information about the crystal setup and collect all the parameters needed to fully define the physical system we are modelling: 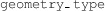 (among 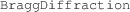, 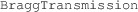, 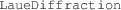 and 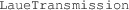), 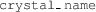 (a string, *e.g.* Si, Ge), 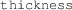 (crystal thickness in SI units [m]), 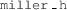, 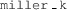, 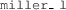 (the Miller indices) and 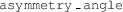 [angle in degrees between the crystal surface and the planes *hkl* as definedby Sanchez del Rio *et al.* (2015 ▸)].

The determination of crystal structure factors (necessary to compute χ_*h*_, χ_−*h*_ and thus *u*_*h*_ and *u*_−*h*_) is not trivial, and requires the list of the crystallographic parameters (basically the cell parameters and a list of the atoms of the unit cell, with their occupation and coordinates). Both 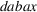 and 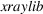 libraries use similar methods that are detailed by Yu *et al.* (2022 ▸). This implementation allows any possible crystal structure. Complex crystals such as alpha-quartz (Sutter *et al.*, 2022 ▸; Sutter *et al.*, 2023 ▸), or YB_66_ (Yu *et al.*, 2022 ▸) are considered. However, some particularities regarding chirality, strong anisotropy or temperature dependence may not be included accurately and are sometimes modelled by phenomenological parameters.

To perform the main calculations (reflectivities, transfer matrices, diffracted photons, *etc*.) several methods in the 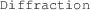 class are used, getting the crystal setup and the photon bunch as inputs. For the moment, only flat perfect crystals are coded (in the 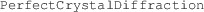 class) which directly implements the formulation and theory in Section 3. For completeness, *crystalpy* also includes the equations of Zachariasen (Zachariasen, 1994 ▸) and can be used instead of the formalism described in this paper. Typical angle or photon scans, as shown in Fig. 2 ▸, are calculated defining a 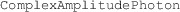 entity for each point, grouping them in a 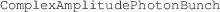 and then calculating the diffraction by the crystal using 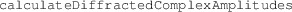.

**Figure 6:**
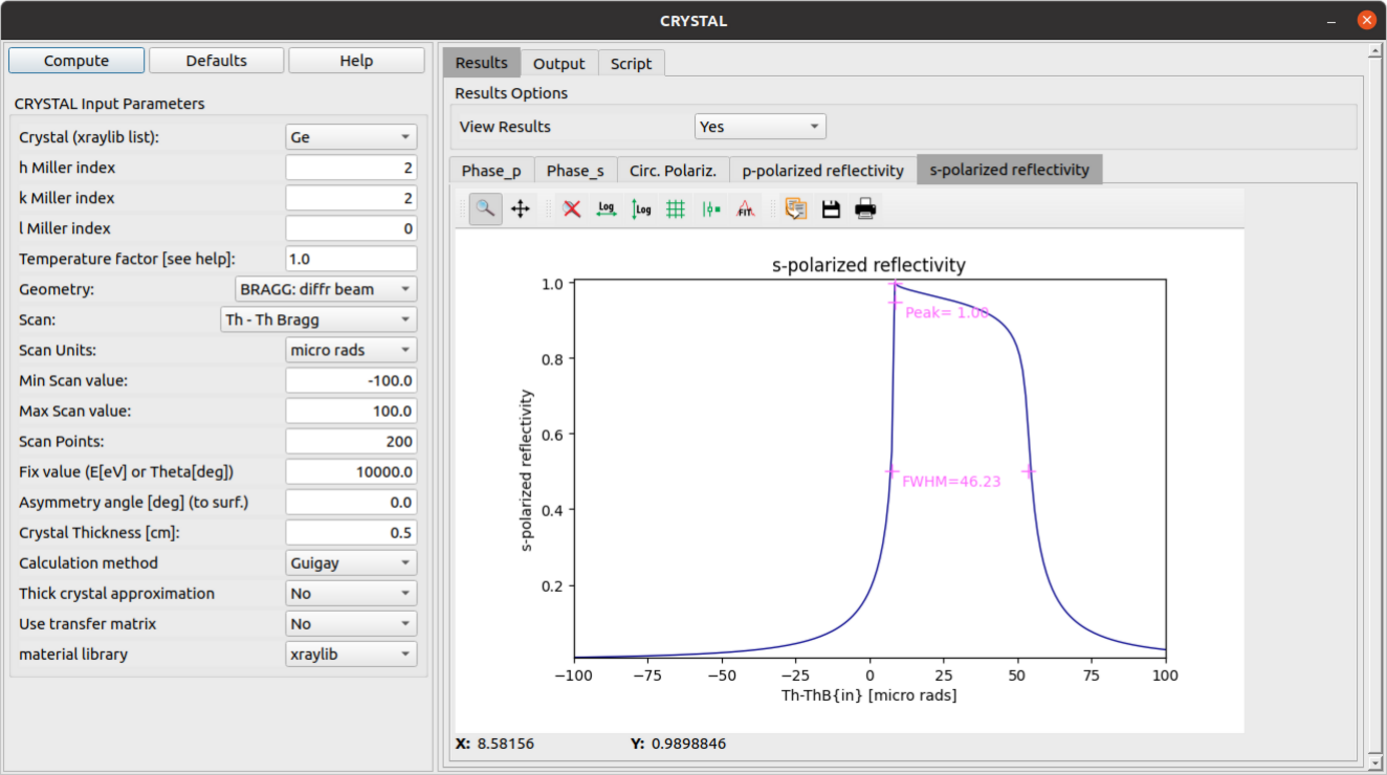
Interactive application for computing the perfect crystal diffraction profiles using *crystalpy* and available in *OASYS*.

A user-friendly application has been written in the *OASYS* environment to compute diffraction profiles using *crystalpy* (Fig. 6 ▸). The applications automatically generates a script that can be used for further batch calculations.

In the ray-tracing *SHADOW4*, all calculations related to crystal optics are delegated to *crystalpy*. Ray-tracing permits simulations of beamline optics including a realistic description of the source. It also allows the simulation of curved crystals, under the assumption that the local reflectivity of the curved crystal is the same as for the flat crystal. This assumption is not always granted and has to be verified before performing ray-tracing simulations including curved crystals.

## 5. Summary and conclusions

We have presented a theoretical and numerical description of dynamical diffraction in perfect crystals. In the first part of this paper we presented a new perspective of the well known dynamical theory of diffraction applied to undeformed perfect crystals. We deduced the equations of diffraction amplitudes (as well as intensity reflectance and transmittance) starting from basic principles via the solution of the Takagi–Taupin equations. We calculated the transfer matrix, useful to compute the diffraction of stacked crystal layers, and also the scattering matrix, of interest for the Bragg case. For completeness, our results are compared with those presented in the well known textbook by Zachariasen (1994 ▸) (see Appendix *C*[App appc]). In the second part we presented *crystalpy*, a software library completely written in Python that implements the theory previously discussed. This open source tool can be used to predict the diffraction properties of any crystal structure, like Si, Ge or diamond typically used in synchrotron beamlines, but also for any other crystal provided its crystalline structure is known. This library is intended to replace multiple scattered pieces of software in packages like *OASYS* (Rebuffi & Sanchez del Rio, 2017 ▸) and is designed to be the kernel of the crystal calculations in version 4 of the *SHADOW* (Sanchez del Rio *et al.*, 2011 ▸) ray-tracing code. The *crystalpy* library and its documentation are available from https://github.com/oasys-kit/crystalpy.

## Figures and Tables

**Table 1:** Correspondences of notation in this work and Zachariasen (1994 ▸)

Zachariasen	This work
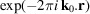	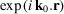
*k*_0_ = 1/λ	*k* = 2π/λ
α_*Z*_	−α
Ψ_0_	χ_0_
Ψ_*H*_	χ_*h*_
*z*	−(λ/π)ω
*X*	(λ/π)*a*
*t*_0_	*t*_c_ = *T*/γ_0_
*m*	*aT*
*c*	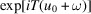

## Footnotes

1 If the approximation in equation (9) were not used, the coefficients of *D*_0,*h*_ in (10*b*) should be multiplied by (1 − α)^−1/2^ ≃ 1 + α/2 +…; since χ_0_, χ_*h*_ and α are much smaller than 1, we neglect the high-order terms.

2 For any point  = , *s* is the path length inside the crystal along : the ray through **r** enters the crystal at the point of coordinates (*s*′_0_, *s*_*h*_, *s*_*t*_) such that γ_0_*s*′_0_ + γ_*h*_*s*_*h*_ = 0, so that *s* = *s*_0_ − *s*′_0_ = *s*_0_ + *s*_*h*_/*b*.

3 These solutions are the Ewald wavefields discussed in detail by Authier (2003 ▸) using the dispersion surface.

4 Our α has the opposite sign to the α defined in equation [3.114b] of Zachariasen (1994 ▸).

5 To see this, suppose  > 0; this implies [equation (27)]  < 0 (note that *b* < 0 for the Bragg case) and [equation (28*a*)]  > 0, therefore [equation (29)]  > 0 if  > 0. Similarly, supposing  < 0, we obtain  > 0 and  < 0, therefore  < 0 if  > 0.

6 The multiplication should be done from bottom to top, *i.e.**M* =*M*_*n*_*M*_*n*−1_…*M*_2_*M*_1_.

## Appendix A Derivation of the TT equations for a rotating perfect crystal

In the ‘rotating crystal mode’, the crystal is rotated around an axis perpendicular to the ‘diffraction plane’ which contains the diffraction vector **h** and the wavevector  of the fixed incident plane-wave in vacuum. The crystal rotation from the exact geometrical Bragg position may be viewed as a special kind of crystal deformation. We propose to use the Takagi–Taupin approach for the deformed crystal to derive the basic results of the dynamic theory for perfect crystal diffraction. The X-ray wavefield inside the crystal is set as

 is the position of the diffraction vector when the crystal is in the exact Bragg condition for the fixed incident wavevector . The vector  =  +  is therefore such that  =  =  = . The Fourier coefficients χ_*h*_ of the perfect crystal susceptibility are replaced by the function , in which  = , with  the displacement field of the deformed crystal. In such conditions, the following form of the TT equations,

is obtained by inserting equation (53) into (1), with the following approximations: the second-order derivatives of*A*_0,*h*_, supposed to be slowly varying amplitudes, are neglected and only the terms containing  in the product χΨ are considered. Introducing oblique coordinates (*s*_0_, *s*_*h*_) in the diffraction plane, along the directions of  and , so that  =  and  = , the TT equations are

Performing the transformation *A*_0_ = *D*_0_ and  = , we obtain

This is identical to equation (10) considering that  = , for which a demonstration follows.

### A1. Demonstration of α = (2/*k*)(∂ϕ/∂*s*_*h*_)

Let  be unit vectors along the fixed directions of  and ; the crystal rotation transforms  into . A position vector  is transformed into . The displacement field is  =  + ;  = . Hence

We note that

and therefore

## Appendix B Solutions of TT equations (17) using the Laplace transform

### b1. Laue solution based on Laplace transform

Let us denote  the Laplace transform of a function *F*(*s*)

Applying the Laplace transform to equations (17) we get

The solutions are

with, as previously defined, *a*^2^ = ω^2^ + *bu*_*h*_*u*_−*h*_, *a* = (ω^2^ + *bu*_*h*_*u*_−*h*_)^1/2^, hence one retrieves the same results of equations (34) using the fact that (*p*^2^ + *a*^2^)^−1^ and *p*(*p*^2^ + *a*^2^)^−1^ are the Laplace transforms of  and , respectively.

### b2. Bragg solution based on Laplace transform

By Laplace transform of equation (17), and calling *r*′ = *B*_*h*_(0), we obtain

or

with (the same as before) *a*^2^ = ω^2^ + *bu*_*h*_*u*_−*h*_. Hence,

*r* and then the reflected and transmitted amplitudes are obtained using the condition *D*_*h*_(*T*) = *B*_*h*_(*T*) = 0. With some calculation, we obtain the results of equations (39).

## Appendix C Equivalence of amplitudes in equation (39) and (34) with Zachariasen’s formulation

The diffracted and transmitted intensities (not the amplitudes) are derived in the book of Zachariasen (Zachariasen, 1994 ▸) (first edition published in 1944). It is shown hereafter that the amplitudes can be easily derived from Zachariasen’s formalism. For that purpose, we use Zachariasen’s notation and equations.

In the Laue case, using equations [3.127] and [3.128] of Zachariasen (1994 ▸), we obtain

Similarly, in the Bragg case, using Zachariasen’s equations [3.127] and [3.135], we obtain^7^

The symbols *c* and *x* are

γ_0_ (γ_*h*_) is the direction cosine of the incident (diffracted) wave and the other quantities are defined as

with *X* = (*q* + *z*^2^)^1/2^,  = , Ψ_*H*_ is the Fourier component of the electrical susceptibility Ψ_0_ and *b* = γ_0_/γ_*h*_ is the asymmetry factor.

It is easy to see that  = ,  = . Introducing the variables  =  and *m* = −2π*k*_0_*Xt*/(2γ_0_), we have

and

Replacing in equation (56) the terms obtained here we finally get

For the Bragg case, we pre-calculate

that we introduced in equation (56) and we finally get

Considering the equivalence of notations between this work and Zachariasen’ book (see Table 1 ▸), we can verify that equations (55) are identical to (34) and, similarly, equations (56) are identical to (39).
